# Spontaneous Regression of a Retinal Astrocytic Hamartoma in an Adolescent With Tuberous Sclerosis Complex

**DOI:** 10.7759/cureus.85633

**Published:** 2025-06-09

**Authors:** Alexander B Dillon, Renae A Tessem, Stacy L Pineles, Tara A McCannel

**Affiliations:** 1 Vitreoretinal Surgery Service, Kaiser Permanente, Vallejo, USA; 2 Vitreoretinal Surgery Service, University of California, Los Angeles (UCLA) Jules Stein Eye Institute, Los Angeles, USA; 3 Ophthalmology Department, David Geffen School of Medicine, University of California, Los Angeles (UCLA), Los Angeles, USA; 4 Pediatric Ophthalmology Service, University of California, Los Angeles (UCLA) Jules Stein Eye Institute, Los Angeles, USA; 5 Ocular Oncology Service, University of California, Los Angeles (UCLA) Jules Stein Eye Institute, Los Angeles, USA

**Keywords:** astrocytic hamartoma, neurocutaneous disorder, ocular oncology, pediatric ophthalmology, retina, tuberous sclerosis

## Abstract

Tuberous sclerosis complex (TSC) is a rare, autosomal dominant syndrome arising from mutations in tumor suppressor genes. Common ophthalmic findings include retinal astrocytic hamartomas (RAHs), retinal achromic patches, and eyelid angiofibromas. In this article, we report a case of an adolescent girl with TSC found to have bilateral RAHs, highlighting the necessity for routine fundus examinations in patients with TSC. Additionally, our case is a rare example of a RAH that spontaneously regressed. RAHs can develop at any age and may rarely threaten vision with potential complications including vitreous hemorrhage, vitreous seeding, exudative retinal detachment, glaucoma, optic nerve invasion, globe perforation, and blindness. If detected early, vision-threatening RAHs may benefit from a variety of therapies, including argon laser photocoagulation, photodynamic therapy, subthreshold micropulse laser photocoagulation, and intravitreal anti-vascular endothelial growth factor (VEGF) agents. A medical history and knowledge of typical manifestations of this phakomatosis and their corresponding physical examination and ophthalmic imaging characteristics aid in the diagnosis. Most retinal astrocytic hamartomas do not cause vision deficit and may be safely observed, and a subset, as seen in this case, may spontaneously regress.

## Introduction

Tuberous sclerosis complex (TSC) is a rare, autosomal dominant syndrome arising from mutations in the *TSC1* or *TSC2* genes [1]. These tumor suppressor genes encode the proteins tuberin and hamartin, respectively, which together comprise an inhibitory heterodimer to the mammalian target of rapamycin (mTOR) cell growth and proliferation pathway [2]. TSC manifestations can arise in any organ system with varied age of onset and severity. Common findings and major diagnostic criteria include skin lesions such as hypomelanotic macules, facial angiofibromas, and shagreen patches; cortical tubers; subependymal nodules and astrocytomas; cardiac rhabdomyomas; angiomyolipomas; and retinal hamartomas [3]. Ophthalmic findings are seen in more than half of TSC patients and include retinal astrocytic hamartomas (RAHs) (present in 36%-50%, with 34%-50% bilateral), retinal achromic patches (a minor diagnostic criterion), and eyelid angiofibromas [4-6]. Less commonly, hypopigmented lesions or hamartomas of the iris and ciliary epithelium, iris and choroidal colobomas, and papilledema in the setting of obstructive hydrocephalus from giant cell astrocytomas have been reported [7-9]. Herein, we present a case showing the spontaneous regression of a RAH in an adolescent girl with TSC.

## Case presentation

A 14-year-old woman with type I diabetes mellitus (T1DM) and TSC was referred to our ocular oncology service after a routine diabetic dilated fundus examination revealed a cream-colored lesion in the inferior periphery of her right eye. At the age of 6.5 months, she presented to an outside hospital with new-onset seizures (myoclonic versus infantile spasms). An electroencephalogram (EEG) showed multifocal independent spike discharges without hypsarrhythmia. An MRI revealed subependymal nodules and cortical tubers. A skin examination revealed a small ash-leaf spot on the right lower extremity, while ophthalmologic examination, echocardiogram, and renal ultrasound were deemed unremarkable. The patient was diagnosed with TSC based on clinical and imaging findings; however, genetic testing was negative for known *TSC1* and *TSC2* mutations. Additionally, neither parent exhibited symptoms of TSC, and there was no evident family history of genetic disorders, suggesting that this may represent a new mutation in the family. At the age of 20 months, a video EEG revealed infantile spasms, which resolved with vigabatrin. By the age of five years, antiepileptic medications had been weaned due to the absence of further seizure activity. She also developed other stigmata of TSC, including multiple bilateral renal cysts, angiomyolipomas, shagreen patches, and facial angiofibromas. At the age of nine years, she was diagnosed with T1DM following presentation with hyperglycemia, elevated A1C, polydipsia, and polyuria.

**Figure 1:**
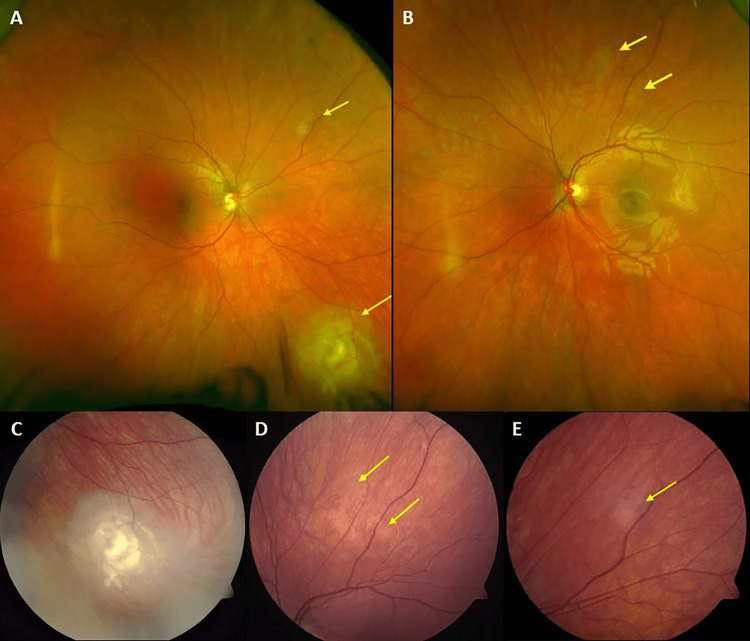
Scanning Laser Ophthalmoscopy (SLO) Images of Multiple Retinal Astrocytic Hamartomas. (A and B) Scanning laser ophthalmoscopy (SLO) images of the right and left eye, respectively, show multiple retinal astrocytic hamartomas. Color fundus photos show (C) the creamy white elevated lesion located inferonasally in the right eye and multiple flat hamartomas (D) in the superior periphery of the left eye and (E) in the superonasal periphery of the right eye.

The patient first presented to our pediatric ophthalmology service at the age of nine years for a routine TSC screening examination with no apparent ophthalmic involvement. Re-evaluation every 1-2 years was unremarkable. She returned to the clinic at age 14 for a routine diabetic dilated fundus examination with no ophthalmic complaints. Her vision was 20/20 in both eyes, and her examination was significant only for a cream-colored lesion with feeding vessels in the inferior periphery of her right eye. Referral to the ocular oncology service and further evaluation were recommended. Multiple cream-colored lesions in the periphery of both eyes were observed (Figure 1A-1E).

**Figure 2:**
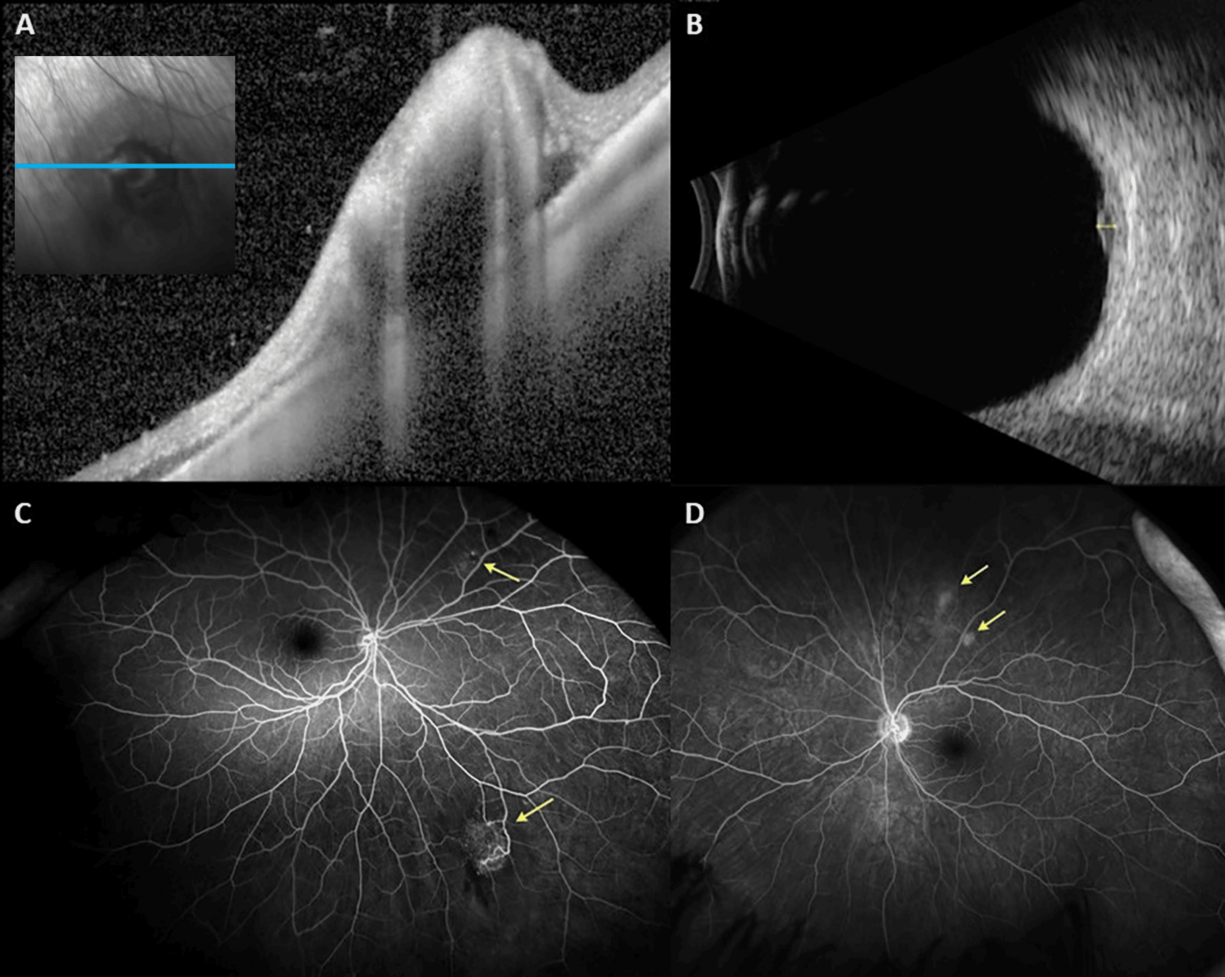
Optical Coherence Tomography (OCT), B-scan Ultrasonography, and Fluorescein Angiography Images of Retinal Astrocytic Hamartomas. (A) A cross-sectional optical coherence tomography (OCT) image of the inferonasal retinal hamartoma in the right eye shows an elevated intra-retinal lesion with trace adjacent subretinal fluid and shadowing artifact. Red-free photography is provided with a marked location corresponding to this OCT section. (B) Ultrasound B-scan of the lesion demonstrates a mixed iso- and hypo-echogenic minimally elevated lesion anterior to the equator at 7:00 inferonasally with an inferonasal thickening height of 1.04 mm and basal diameter of 4.41 × 4.83 mm. (C and D) Ultra-widefield fluorescein angiography of the right and left fundus, respectively, show staining of the lesions and highlight the hyperfluorescent feeder vessels associated with the inferonasal lesion in the right eye.

**Figure 3:**
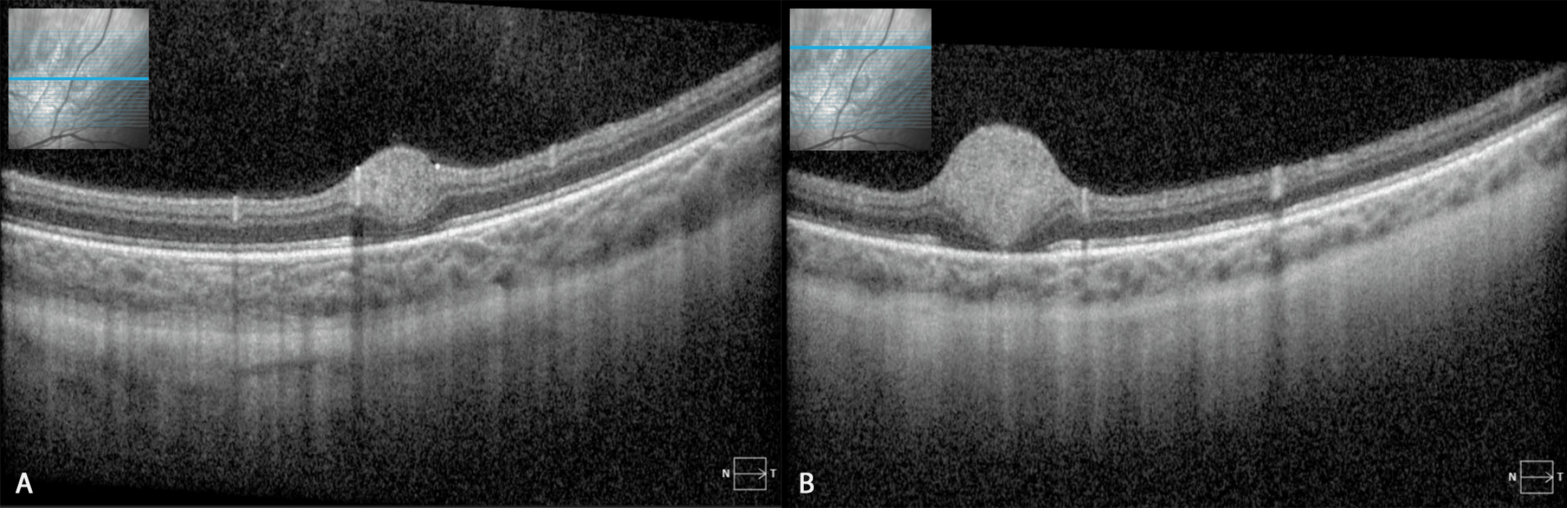
Optical Coherence Tomography (OCT) of Multiple Flat Retinal Astrocytic Hamartomas. (A and B) Cross-sectional optical coherence tomography (OCT) images of the superonasal periphery of the left eye show multiple flat hamartomas. Red-free photography is provided with a marked location corresponding to each OCT section.

**Figure 4:**
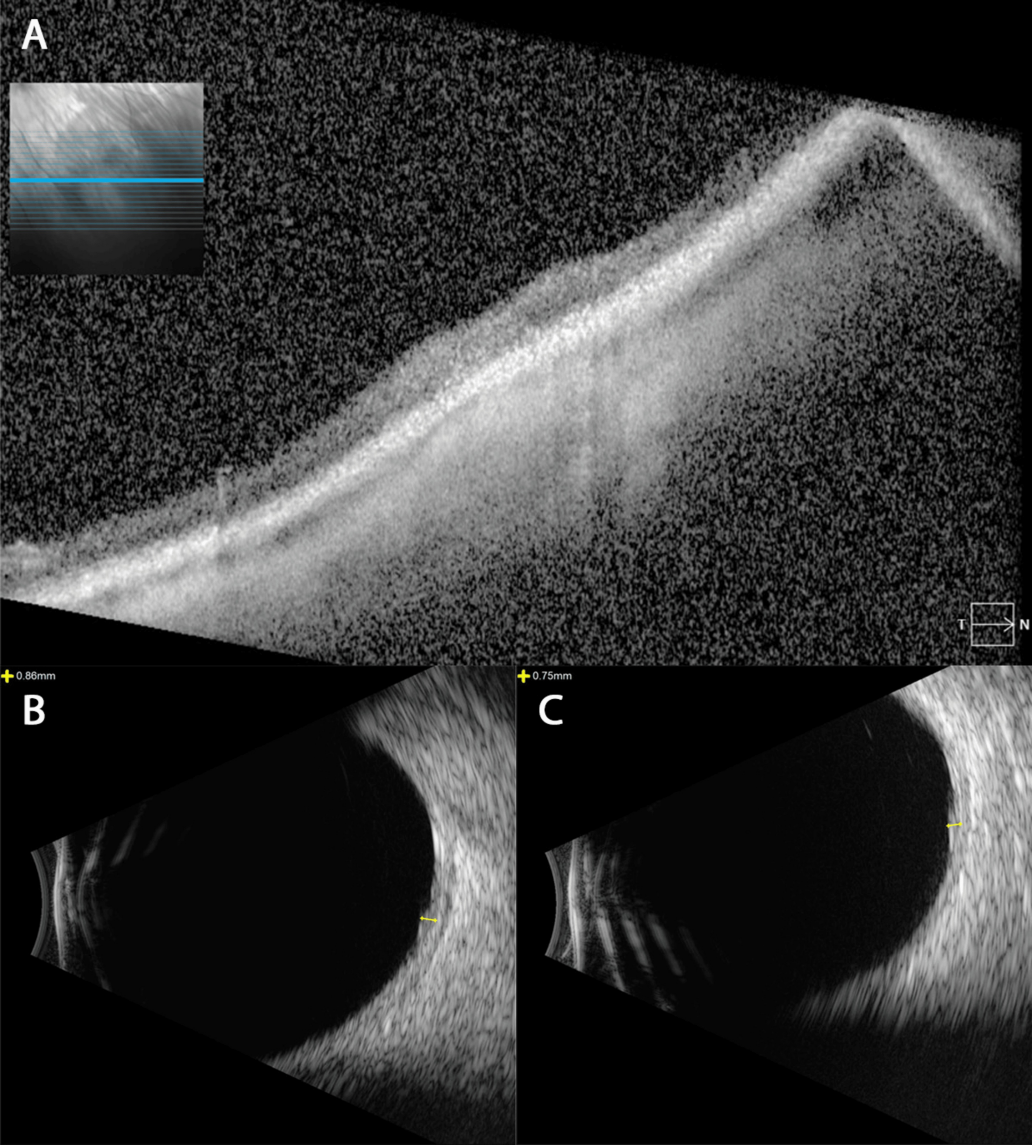
Optical Coherence Tomography (OCT) and B-scan Ultrasonography of Retinal Astrocytic Hamartoma of the Right Eye That Spontaneously Regressed. (A) A cross-sectional optical coherence tomography (OCT) image of the regressed inferonasal retinal hamartoma of the right eye. (B and C) B-scan ultrasonography of the right eye showed barely detectable foci of retinal thickening anterior to the equator at 4:00 inferonasally and near the equator at 7:30 inferotemporally. The largest lesion of the right eye now showed an inferonasal thickening height of 0.75 mm (previously 1.04 mm) and an inferotemporal thickening height of 0.86 mm (not noted previously).

Optical coherence tomography (OCT) imaging revealed a mixed hyper- and hyporeflective intra-retinal lesion with trace abutting subretinal fluid in the right eye (Figure 2A). B-scan ultrasonography showed a minimally elevated lesion anterior to the equator at 7:00 inferonasally with an inferonasal thickening height of 1.04 mm and basal diameter of 4.41 × 4.83 mm (Figure 2B). Fluorescein angiography showed hyperfluorescent feeder vessels and lesion staining (Figure 2C, 2D). The other lesions were flat (Figure 3A, 3B). The dilated fundus examination and imaging findings were consistent with multiple retinal astrocytic hamartomas. Observation with re-evaluation in 2-3 months was recommended. The patient was lost to follow-up; however, when she returned to the clinic more than two years later, the largest lesion in the right eye had regressed (Figure 4A). B-scan ultrasonography of the right eye showed barely detectable localized elevations anterior to the equator at 4:00 inferonasally and near the equator at 7:30 inferotemporally. The largest lesion of the right eye had a height of 0.75 mm (previously 1.04 mm) and an inferotemporal thickening height of 0.86 mm that was not previously noted (Figure 4B, 4C). Observation with re-evaluation in 12 months was recommended.

## Discussion

Retinal astrocytic hamartomas are a hallmark of TSC, though they can also be found in isolation or in association with neurofibromatosis, and hence are not pathognomonic for this condition. Most retinal hamartomas in TSC patients are flat, translucent, yellow-white lesions with blurred margins, without calcifications, and are found most often at the distal arcades [8]. A subset is elevated with a classic multilobular “mulberry” appearance and calcifications and has a proclivity for the posterior pole. Transitional or “mixed” hamartomas, such as the elevated lesion in our patient, have features of both types [5,8]. Most of these lesions remain stable over time, though a subset may grow, calcify post-utero, and occasionally arise from previously healthy-appearing retina [7].

While a baseline ophthalmologic evaluation is recommended for all patients with TSC, bilateral RAHs were identified in our patient more than 13 years after the original TSC diagnosis, highlighting the utility of routine funduscopic examination in patients with TSC [3]. As of 2021, the updated TSC diagnostic criteria and surveillance and management recommendations suggest annual ophthalmic evaluation in TSC patients, regardless of whether they exhibited visual symptoms at baseline [3]. These serial examinations can aid in monitoring for new or changing lesions and help with the early detection and treatment of rare complications.

While morbidity arising from RAHs is fortunately rare, vitreous hemorrhage, vitreous seeding, exudative retinal detachment, glaucoma, optic nerve invasion, excessive growth with globe perforation, and painful blindness requiring enucleation have all been described [10-12]. Notably, of the 11 cases of TSC-associated retinal astrocytic hamartomas managed by enucleation that have been reported, the age at enucleation ranged from one to 27 years, with the majority at age 10 years or less. The majority of the causative lesions were peripapillary in location, while other retinal hamartomas noted elsewhere in the affected eye in a subset of these patients were observed to be stable over time [12-20]. Based on these observations, smaller and more peripheral RAHs, such as those seen in our case, may be at lower risk for progressive growth requiring enucleation [12]. In contrast, our case is a rare example of a RAH that spontaneously regressed. Although rare, a handful of cases have documented the partial or full regression of isolated and TSC-associated RAHs, predominantly in young children within about nine months to two years following the identification of the lesions [21-24]. RAH-associated macular subretinal fluid has also been reported to spontaneously regress within a month of presentation in a handful of reported cases [25-28]. Given that most RAHs are stable and do not impair vision and a subset may spontaneously regress, as observed in our case, observation is recommended in uncomplicated cases [22].

For patients with persistent vision deficit from rare, sight-threatening RAHs, a variety of treatment modalities have been suggested to be effective. Argon laser photocoagulation has been employed with variable success, though the risk of collateral thermal retinal destruction and secondary choroidal neovascularization may limit first-line use for macular lesions [29,30]. Photodynamic therapy has repeatedly proven successful in facilitating the resolution of submacular fluid, inducing tumor regression, and improving visual acuity in these patients [31-33]. Subthreshold micropulse laser photocoagulation with or without the use of intravitreal anti-vascular endothelial growth factor (VEGF) agents may also be effective in addressing symptomatic macular lesions [34]. Further support for the consideration of anti-VEGF for RAH-associated intra- and subretinal fluid was lent by elevated VEGF levels in the vitreous [28], RAH tissue and associated neovascular membrane [35], and multiple reported patients with improvement following anti-VEGF adjunctive therapy [35-37].

While mTOR inhibitors such as everolimus and sirolimus have been shown to reduce the size of retinal astrocytic hamartomas, in light of the indolent nature of the majority of the lesions studied, most patients have not benefited visually [38-41]. In one case of a 13-month-old boy with TSC and a retinal hamartoma with associated macula-involving exudative retinal detachment refractory to laser photocoagulation and intravitreal bevacizumab, the initiation of oral everolimus resulted in a reduction in the size of the causative lesion, the resolution of the subretinal fluid, and improvement in macular exudates at 13-month follow-up [42].

Intraocular surgery is not typically necessary, though it may be beneficial in the rare cases of non-clearing secondary RAH-associated vitreous hemorrhage or proliferative retinopathy with visually significant tractional membranes [10,35,43,44].

## Conclusions

Tuberous sclerosis complex-associated retinal astrocytic hamartomas are a common facet of the tuberous sclerosis complex constellation and warrant routine fundus examination in TSC patients, as they can arise throughout life and may, albeit rarely, threaten sight and the eye. Fortunately, multiple treatment modalities may successfully address visually significant lesions, especially if detected early. A medical history and knowledge of typical manifestations of this phakomatosis and their corresponding physical examination and ophthalmic imaging characteristics aid in the diagnosis. Most retinal astrocytic hamartomas do not cause vision deficit and may be safely observed, and a subset, as seen in this case, may spontaneously regress.
